# Understanding the China-Tanzania Malaria Control Project: lessons learned from a multi-stakeholder qualitative study

**DOI:** 10.3389/fpubh.2023.1229675

**Published:** 2023-09-21

**Authors:** Zhishan Sun, Hui Zhou, Fumin Chen, Shenning Lu, Huan Liang, Erya Wan, Zecheng Tao, Hanqing Zhao, Xiaonong Zhou, Fan Yang, Duoquan Wang, Xiaoxi Zhang

**Affiliations:** ^1^School of Global Health, Chinese Center for Tropical Diseases Research, Shanghai Jiao Tong University School of Medicine, Shanghai, China; ^2^Institute of One Health, Shanghai Jiao Tong University, Shanghai, China; ^3^School of International and Public Affairs, Shanghai Jiao Tong University, Shanghai, China; ^4^National Institute of Parasitic Diseases at Chinese Center for Disease Control and Prevention (Chinese Center for Tropical Diseases Research), NHC Key Laboratory of Parasite and Vector Biology, WHO Collaborating Centre for Tropical Diseases, Shanghai, China; ^5^School of Public Health, Shanghai Jiao Tong University School of Medicine, Shanghai, China; ^6^Institute of Population Research, Peking University, Beijing, China

**Keywords:** qualitative study, malaria control, global health intervention, China, Tanzania

## Abstract

**Background:**

Tanzania is among the countries with the highest malaria cases and deaths worldwide, where vulnerable populations have been severely affected due to poverty and weakness in health system and infrastructure. The China-Tanzania Malaria Control Project (the Project) was a two-phase global health intervention project implemented between 2015 and 2021 that aimed to transfer project-designated intervention experience in malaria elimination to the Tanzanian health system. This study aims to identify the barriers and facilitators encountered during the Project and to improve our understanding of the emerging phenomenon of South-South global health collaboration.

**Methods:**

We conducted thematic analysis of qualitative data collected from a purposive sample of 14 participants from multiple stakeholders including project management office, project implementation agency, funding partners and external evaluators of the Project. A conceptual framework was developed to construct the interviews guides. The interviews were transcribed verbatim, crossover checked, translated into English, and analyzed with NVivo 12.0. We conducted the open coding followed by the axial coding based on the Grounded Theory to generate themes and subthemes, and identified key influencing factors that aided or hindered the malaria control in Tanzania.

**Results:**

The findings suggested that malaria control strategies should largely be tailored due to varied socioeconomic contexts. The perceived enablers in practice include project-designated intervention experiences and technologies, professional and self-learning capabilities of the implementation team, sustainable financial assistance, and support from the international partners. The barriers include the shortage of global health talents, existing gaps to meet international standards, defects in internal communication mechanisms, inadequacy of intergovernmental dialogue, and limitations in logistical arrangements. A checklist and policy implications for China's future engagement in malaria control in resource-limited settings have been proposed.

**Conclusions:**

The initiative of Health Silk Road has generated strong global interest in promoting development assistance in health. In the hope of generalizing the evidence-based interventions to high malaria-endemic countries in Africa, the need for China to carefully face the challenges of funding gaps and the lack of support from recipient governments remains ongoing. It is recommended that China should form an institutionalized scheme and sustainable funding pool to ensure the steady progress of development assistance in health.

## 1. Introduction

Malaria poses a threat to global health. It has been estimated that 247 million malaria cases occurred in 2021 in 84 malaria-endemic countries, an increase from 245 million in 2020 (1). The World Health Organization (WHO) African Region accounts for 80% of all malaria cases worldwide (2). Tanzania is one of the 11 countries affected by 70% of the global malaria burden (3). Substantive efforts have been made to promote bilateral and multilateral cooperation among international institutions and to provide funds to control malaria transmission in resource-poor regions in Tanzania (4). As a result of a combination of preventative and therapeutic strategies, the prevalence of malaria parasites in the country has decreased by 50% over the past decade (5). Despite this reduction, malaria remains a major cause of death and morbidity across all age groups in Tanzania (6).

In spite of being afflicted by the scourge of malaria 77 years ago with 30 million cases annually, China has been certified malaria-free on June 30, 2021 (7). The achievement of malaria control of China was partly due to the innovation of artemisinin and the development of the 1-3-7 strategy (reporting cases within 1 day, completing case investigation within 3 days, completing foci investigation and transmission interventions within 7 days) (8). In order to transfer the successful experience of China in malaria control and contribute to global malaria elimination, China inaugurated China-Tanzania Malaria Control Project (the Project, 2015–2021), which has been divided into two phases including Phase I (China-Tanzania Pilot Project, 2015–2018) and Phase II (China-Tanzania Demonstration Project, 2018–2021) (3, 9). Guided by the WHO's initiative of T3 strategy (Test, Treat, Track) (10), this project seeks to find solutions to adapt the 1-3-7 strategy, which has been demonstrated effective in China's context, into moderate to high transmission setting in Tanzania.

In Phase I, funded by the Department for International Development (DFID) in the UK, the project implementation agencies (PIAs), including the National Institute of Parasitic Diseases (NIPD) at Chinese Center for Disease Control and Prevention in cooperation with the Ifakara Health Institute (IHI) in Tanzania, proposed and tested an integrated package of intervention measures called 1,7-malaria Reactive Community-based Testing and Response (1,7-mRCTR) at two intervention sites in the Rufiji Region, South Tanzania (11). Besides integrated strategies in case mangement and vector control, 1,7-mRCTR requires health facilities to report confirmed malaria cases within 24 h and to provide intensive treatment to pandemic villages within 1 week (8). Comparing with 1-3-7 strategy, 1,7-mRCTR is appropriate for areas with high incidence rates and limited medical resources, with the focuses on treating people in endemic areas at the community level (12). Subsequently, in order to validate the outcomes of the strategies used in Phase I to guide further evidence-based policy recommendations, Phase II was initiated and expected to scale up the 1,7-mRCTR from two intervention sites to six in Tanzania, with the funding from the Bill and Melinda Gates Foundation (BMGF) (13, 14).

Given the extensive resources and abundant experience gained, it has become crucial to identify the enablers of and challenges affecting both the implementation process and outcomes of the Project in Africa. As previous studies mainly focused on second-hand documents explored using narrative review (15), little qualitative evidence about South-South multilateral projects on malaria control in Africa has yet been produced. This study proposes qualitative insights based on in-depth interviews with various stakeholders actively engaged in both Phase I and Phase II, so as to elicit information regarding ways of transferring 1,7-mRCTR tailored to malaria control to Africa, and inform future multilateral implementation projects for scaling up effective strategies in malaria control in resource-limited areas.

## 2. Methods

### 2.1. Study participants

In this study, purposive sampling was used to recruit stakeholders involved in the Project across four categories: PIAs, project management offices (PMOs), finance funders, and external evaluators. The members of PIAs consisted of experts from the NIPD and IHI. PMOs was a center of the China's National Health Commission that was responsible for supervising fund use and project implementation during Phase I.

The eligibility criteria for the study participants were as follows: (i) highly responsible for or involved in the Project, including policy formulators, management or implementation personnel, health care providers, project funders, etc; (ii) engagd with the Project for at least 1 year; (iii) in mid or late stage of career. Individuals who met all criterias were included in our study. Interviewers then contacted individuals who had participated in the project by phone or email to determine their willingness to be interviewed and to explain the purpose of the study.

### 2.2. Data collection

**Figure 1:**
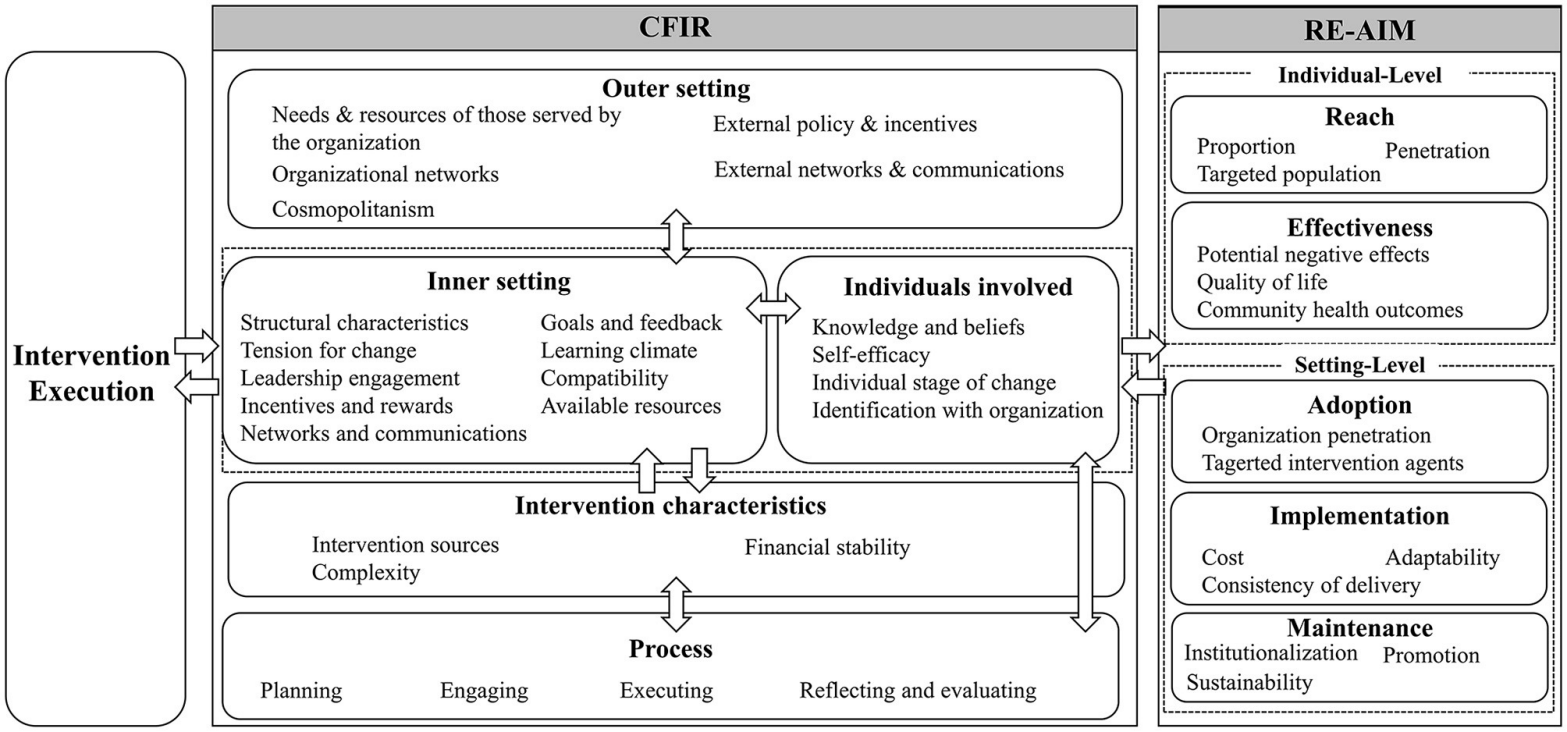
Theoretical framework.

An conceptual framework combining the Consolidated Framework for Implementation Research (CFIR) (16) and the Reach, Effectiveness, Adoption, Implementation, and Maintenance (RE-AIM) framework (17) was used in this study (Figure 1), to guide the development of interview instruments and interpretation of results.

Semistructured interview guides (Supplementary material 1) were designed and tailored to different types of stakeholders. A pilot interview was held to refine the interview guides. The final version of the interview guides was formulated after discussion with malaria prevention experts and health policy specialists.

All in-depth interviews were held online by a group of researchers in Chinese or English, and they featured a duration of 50–60 min each. All researchers have undergone qualitative research training by qualitative research experts over 3 days. The research team did not have any contact with the interviewees prior to obtaining informed consent. Each interviewee signed an informed consent form before the interview. To build rapport with all participants, interviewers explained the reason for the interview at the beginning of the interview. The interviews were conducted from August 2022 to March 2023. The interviews were video recorded and transcribed verbatim for analysis. Each interview transcription was double-checked by a second researcher. The interview process is shown in Table 1.

**Table 1:** Interview process and category of interviewees.

**Interview time**	**No**.	**Interviewee code**	**Category**	**Involvement in Phase I & II**	**Interviewer**
08/20/2022	1	PIA-1	Implementer	1	Zhishan Sun
08/20/2022	2	PIA-2	Implementer	1	Fumin Chen
08/20/2022	3	PIA-3	Implementer	1	Fumin Chen
08/22/2022	4	Funder-1	Funder	2	Erya Wan
08/23/2022	5	PMO-1	Manager	1	Xiaoxi Zhang
08/24/2022	6	PIA-4	Implementer	1	Fumin Chen
08/25/2022	7	PIA-5	Implementer	Both	Hui Zhou
08/25/2022	8	PIA-6	Implementer	1	Zhishan Sun
08/25/2022	9	PIA-financial	Implementer	1	Fumin Chen
08/27/2022	10	PIA-7	Implementer	Both	Erya Wan
08/29/2022	11	PMO-2	Manager	1	Xiaoxi Zhang
02/20/2023	12	Evaluator-1	Evaluator	Both	Hui Zhou
03/17/2023	13	Evaluator-2	Evaluator	2	Xiaoxi Zhang
03/17/2023	14	Evaluator-3	Evaluator	2	Xiaoxi Zhang

We stopped recruiting more participants once data saturation was reached (18). After the fourteenth interview, the researchers reached a consensus concluding that saturation had been reached.

### 2.3. Data analysis

Drawing on the Grounded Theory methodology, we conducted open coding, axial coding and then focused coding to allow themes and subthemes to emerge from the data (19). Codes were named either from the transcription or based on constructs drawn from the conceptual frameworks. Researchers used NVivo12.0 software during the coding of all interviews. In coding, we specifically focused on barriers to and enablers of the Project. Three researchers (ZS, HZ, FC) first coded the data independently and then discussed and reached a consensus regarding the codebook (Supplementary material 2). We followed the Consolidated Criteria for Reporting Qualitative Research (COREQ) during the analysis and reporting of our study (20) (Supplementary material 3).

## 3. Results

### 3.1. Overview

We conducted 14 in-depth interviews with eight experts from PIAs, two from PMOs, one from project funder, and three external evaluators. The distribution of interviewees across stakeholder type and demographic characteristics is summarized in Table 2. Our study yielded salient themes and subthemes pertaining to the enablers and challenges associated with the Project (Table 3).

**Table 2:** Characteristics of participants.

**Variables**		**Implementer (PIA)**	**Funder**	**Manager (PMO)**	**Evaluator**
Gender	Male	7	0	1	3
	Female	1	1	1	0
Education	Bachelor	3	0	1	0
	Master	2	1	1	0
	Doctor	3	0	0	3
Title	Researcher	4	0	0	3
	Researcher Associate	3	0	0	0
	Other	1	0	2	0
Country	China	8	1	2	0
	Others	0	0	0	3
Profession	Technical	7	0	0	3
	Financial	1	0	0	0
	Management	0	1	2	0

**Table 3:** Overview of key themes.

**Themes**	**Subthemes**
1. Overview	1.1. Phase I (the Pilot Project) 1.2. Phase II (the Demonstration Project)
2. Identified setting variations	2.1. Health system profile 2.2. Epidemiologic profile 2.3. Public awareness and health literacy
3. Perceived enabling factors in practices	3.1. Localization of experience in malaria control 3.2. Professional and self-learning capabilities of the implementation team 3.3. Sustainability in multisource funding 3.4. Maintenance of international partnerships
4. Perceived absent reinforcing factors in implementation	4.1. Cultivation of the global health workforce 4.2. Alignment with international standards 4.3. Internal coordination among multiple stakeholders 4.4. Adaptation to evolving environment
5. Perceived challenges in future project design	5.1. Inadequacy of intergovernmental dialogue 5.2. Limitations of the logistical arrangements

Most of our interviewees participated in both Phase I and Phase II and they emphasized the importance of highlighting the difference between Phase I and Phase II. The comparison of key characteristics of Phase I and Phase II is summarized in Table 4.

**Table 4:** Comparison of key characteristics of Phase I and Phase II.

**Themes**	**Phase I (Pilot Project)**	**Phase II (Demonstration Project)**
Period	2015–2018	2019–2022
Project nature	Subproject of GHSP	Independent project
Leadership type	Government-led	Non-state actor-organized
Key stakeholders	UK: DFID; China: NHC, NIPD; Tanzania: IHI	BMGF, NIPD, IHI, WHO
Decision-making process	Vertical-Strategic Oversight Committee oversight and led, PMO managed and assessed, PIAs and IHI implemented and reported	Horizontal-Addressed and reached decisions through the Coordination Management Committee (CMC)
Funding source	DFID	BMGF
Funding distribution	First advance payment and reimbursement of actual costs afterward (paid in installments)	Paid in lump-sum
Evaluation	Third-party evaluation including a baseline survey, and mid-term and final evaluations	Full-process external evaluation from cost-effectiveness evaluation to epidemiological assessment

Phase I served as one of the pilot projects funded by the 7-year “Global Health: Science and Practice (GHSP)”. Led by the Chinese and UK governments, Phase I maintained a rather vertical decision-making flow with the top-down financial flow and bottom-up report line. The funding was subject to the allocation of GHSP, in an approach of the first advance payment and the actual costs reimbursed afterward. The payments in installments were assessed and distributed every quarter. Phase II instead was an independent project among BMGF, NIPD, and IHI. It implemented a flat organizational structure with a horizontal decision-making platform, known as Coordination Management Committee (CMC). The funding distribution is a lump-sum system. Compared to Phase I, Phase II added a more comprehensive external evaluation that includes a cost-effectiveness analysis to collect full-process evidence.

### 3.2. Identified setting variations

The foundation for providing effective development assistance in health (DAH) is the understanding of the local characteristics of the political system, public health system, epidemiological design, health policies, and public health awareness in intervention settings. The degree of knowledge of the intervention context directly influences the implementing group's choice of proper intervention strategies.

#### 3.2.1. Health system profile

The study found that the limited budget allocated to public health in Tanzania resulted in inadequate infrastructure and resources for health care delivery. Participants further observed that the high burden of infectious diseases, especially malaria [90% of malaria cases occur in sub-Saharan Africa (4)], resulting in competing priorities for health workers and financial resources. Participants also reported weak capacity in infrastructure, e.g., the lack of internet access. The national health institutions thus lacked electronic surveillance capabilities.

The establishment of the National Malaria Control Program (NMCP) dates back to 2007 through Tanzania's Ministry of Health and Social Welfare. Its primary objective is to bolster Tanzania's malaria control capabilities significantly. In close collaboration with WHO country office and the Swiss Tropical and Public Health Institute, the Tanzanian government has crafted the National Malaria Strategic Plan (NMSP) 2021–2025 for recent action plan. This strategic blueprint outlines a comprehensive package of intervention measures encompassing vector control, case detection, treatment, and prevention programs. Nonetheless, the development of explicit protocols and prerequisites for routine malaria prevention in regions with high incidence necessitates the accumulation of on-site empirical evidence (21, 22).

Tanzania has a complete three-level health system, extending from the country to the region, to the district, and then to the village. However, grassroots-level health facilities in Tanzania are usually understaffed, resulting in the ineffective implementation of interventions for the rural areas. The community-based mobile testing units introduced by the 1.7-mRCTR present a viable avenue to access remote locations and extend services to marginalized populations.

Participants noted that the 1.7-mRCTR initiative effectively operationalized the principles set forth in the T3 initiative, rendering it a practical model for on-ground malaria control efforts. The various interventions in the Project complement the NMCP strategy and facilitate the practical implementation of malaria control measures in the community level.

*Due to limited funds, ITNs can only be distributed to children under five or nine and pregnant women in the community, not to everyone (Evaluator-2, Universite des Montages, Cameroon)*.

*One of the officials told me that we were very happy to sit in the office and see the dashboard, to see videos that show the impact of the intervention. The limitation was that you need to have an internet bundle. Now, an internet bundle is still very expensive in Africa…So, they should have data managers (Evaluator-2, Universite des Montages, Cameroon)*.

#### 3.2.2. Epidemiologic profile

Participants reported great differences in malaria epidemics between China and Tanzania. Due to climatic characteristics, the activity patterns, density, and species of the vectors in Tanzania were different from China.

*There are very many species of Anopheles in Africa. All year round, regardless of the dry or rainy season, the corresponding vector species can survive well in the local environment. They are spreading malaria all year round (PIA-1, NIPD, China)*.

#### 3.2.3. Public awareness and health literacy

Participants noted that the local population had weak perceptions of the severity of malaria due to the high prevalence of malaria infection and that the population exhibited low adherence to the use of malaria medication due to the financial burden of purchasing such medication.

Participants reported that, the differences in the working patterns and attitudes between local health workers and Chinese on-site workers, have affected the efficiency of implementation measures during the initial stage of the Project.

*We ask them, for example, how do you treat yourselves against malaria … But others are telling us, you know what, we can just go for coconut juice. We mix it with the lemon, and when we boil it, we drink and get it OK (Evaluator-3, WHO, Tanzania)*.

### 3.3. Perceived enabling factors in practice

#### 3.3.1. Localization of experience in malaria control

Previous experience with malaria control in China provided the basis for the intervention programme. Considering the feasibility of the project in the pilot area, Chinese experts conducted surveys to investigate the local political, social, economic, cultural, and medical conditions in Tanzania and maintained close communications with the Tanzania IHI.

Based on the commonalities among countries in the Global South, the intervention team developed a strategy of integrated control—vector control, case management, mass mobilization, and CDC system construction—to construct an effective and scalable malaria control system in the intervention areas.

Given the epidemiological characteristics and health system capacity in Tanzania, participants reflected on the process by which PIAs could collaborate on the transformation of the 1-3-7 strategy to 1-7 mRCTR to adapt to the local reality. Through the use of an in-time mobile malaria case monitoring system, which combined mass screening and treatment of key infected populations in each village, “peak clipping” in high malaria incidence areas and high incidence periods was ensured. Participants reported that the temporary regional clearance of malaria was achieved within seven days, thus curbing the widespread of malaria to a larger extent.

*Why 1-7? It was a product of trial and error. When we were building mobile malaria testing stations, we found that it was more appropriate to finish region-wide testing within seven days given the human and medical resources, adding on local fixed testing stations (PIA-3, NIPD, China)*.

*We realize that the T3 will not work. We even went for one 1-3-14 initially in Phase II, and we realized that the 1-3-7 strategy was a bit hard to implement, and this was with the various interactions and the various implementation exercises. We realized that the 1,7 worked easily and was easily accommodated by the local community across various districts and had more impact (Evaluator-2, Universite des Montages, Cameroon)*.

Drawing on the experience of barefoot doctors in rural China, implementers in Tanzania recruited local medical professionals from China who were responsible for implementing malaria-related training with the goal of developing a stable cadre of local primary care workers during the intervention. Participants noted that, unlike research-based health interventions, the capacity-building activities ensured the self-sustainability of the local health system.

The evaluation experts noted that the interventions significantly reduced the local incidence of malaria, eased the disease burden on large hospitals, and relieved the strain on medical resources, as many malaria patients were treated at health facilities at the local level. The project also had a profound social impact, greatly improving the quality of life and wellbeing of the local people.

*Because malaria cases have been treated in the communities, that allows them to have enough time to take care of other patients... I think especially for the community worker. It also provided them with the opportunity to be employed during the life cycle of the project... Many students who were suffering from malaria could go to school. I think the programme actually had a great impact at the community level (Evaluator-3, WHO, Tanzania)*.

To improve the knowledge of malaria control among local residents, the implementation team organized several community activities to promote health education during festivals such as International Malaria Day and National Day. Additionally, participants emphasized the fact that they integrated the dissemination of knowledge regarding malaria into the local culture in various ways, such as through soccer games, theater dramas, and religious activities, with the goal of improving the health literacy of local residents regarding malaria.

*We will design some logos and promotional products according to local customs and cultural preferences. This is also one of the things they like very much locally (Funder-1, BMGF, China)*.

#### 3.3.2. Professional and self-learning capabilities of the implementation team

The Chinese technical experts had professional backgrounds in medicine, economics, management, and other related fields as well as various degrees of relevant work experience. Many of the implementers had engaged deeply and made significant contributions to the elimination of malaria in China. The professionalism of the team ensured the scientific validity and effectiveness of the intervention measures.

The intervention team exhibited strong self-learning capabilities. The Phase II implementation process drew on many lessons learned from Phase I. External audits and cost-effectiveness evaluations were introduced, and feedback from local people was considered in the refinement of intervention measures.

Participants reported that the immediate and standardized communication mechanism ensured no information gap between the onsite PIA and PMO, and this communication flow facilitated process monitoring and project supervision and ensured efficient and effective project outcomes.

*PIA conducted monthly work progress meetings and made appropriate modifications to the following month's schedule, while quarterly financial flow meetings were conducted at the PMO level to confirm work progress and reimburse expenses (PIA-financial, NIPD, China)*.

#### 3.3.3. Sustainability in multisource funding

As a UK ministerial department responsible for international development, DFID funded the Phase I, in line with its strategy to aid China in enhancing its engagement in global health collaboration. Having witnessed the accomplishment in Phase I, BMGF became the funder to maintain and expand the interventions in other regions in Tanzania in Phase II. Through continuous efforts in scientific research and policy advocacy, the effectiveness and visibility of the project have been demonstrated, which leads to the follow-up financial support from the United Nations Peace Fund, to further expand the scope of the interventions to encompass four African countries. The supports of the fundings from multiple sources, have enabled the achievement of the long-term impact of the project.

*We all went on-site to examine the final project results, including the academic articles they published. Some of the interventions have really been continuously developed and implemented, and IHI Tanzania is making a very serious effort to maintain these results. In terms of nonprofit research, I think it should be positive for the local community in general (PIA-financial, NIPD, China)*.

*BMGF has seen 70 years of anti-malaria achievements and scientific research in China and hopes to support China in stepping up and playing a more important role in global health governance (Funder-1, BMGF, China)*.

#### 3.3.4. Maintenance of international partnerships

Participants reported that the WHO's declaration of malaria elimination for China in 2020 was a major boost to the implementation of the Project. The Project was supported by international recognition of China's malaria control achievements over the years. Participants also noted that the institutional establishment of the China International Development Cooperation Agency after Phase I indicated a process by which China's global health governance was institutionalized, reflecting the Chinese government's determination to promote DAH.

Participants noted that the longstanding friendly relationship between China and Africa also helped Chinese implementers integrate themselves into the lives of the local population more effectively and contributed to the high acceptability of the Project among the public.

*The government, the head of the programme (NMCP), said that they all have shown commitment to support IHI and supported the implementation of activities that would help promote malaria control. Also, at least, there's so much assurance that if there's evidence enough to allow us to scale up the activities, there will be no obstacle because the government has said so (Evaluator-3, WHO, Tanzania)*.

### 3.4. Perceived absent reinforcing factors in implementation

#### 3.4.1. Cultivation of the global health workforce

The Project lacked a project manager with professional management experience. A participant noted that the implementers were all dispatched by the NIPD, a government-led organization specialized in tropical disease control in China, which had sufficient malaria control capacity and knowledge but lacked management skills, experience, and professional sensitivity with regard to project management. The lack of managers in the field led to negligence in the project schedule.

Participants reported language barriers in the process of communicating with local staff or residents, which affected the efficiency of project implementation to some extent. To improve the technical and linguistic skills of the project team members, the Chinese and Tanzanian experts engaged in mutual exchange and training activities in data analysis, project monitoring, etc.

*There are many opportunities for Tanzanian and Chinese staff to learn from and communicate with each other, including some graduate supervisors' mutual employment systems among experts. That is to say, they can come to NIPD as graduate supervisors, and we can also go to their institutions as graduate supervisors (PIA-4, NIPD, China)*.

#### 3.4.2. Alignment with international standards

The management team for the Project lacked the necessary knowledge for the international standards of external assessment, proper third-party auditing, ethical review, etc. During the global health project, it is essential to consider the alignment with the requisites of international standards in the early stage of project design, especially in the procurement of supplies and medications, the collection of data and samples, the ethical considerations, etc. The failure to have adequate knowledge of international norms in advance has caused extra cost in time and resources of the Project.

Participants noted that the Phase I budget did not introduce due diligence and post-audit mechanisms, which led to the failure to identify management issues such as inconsistencies between the two fiscal years, leading to a significant depletion of on-site intervention time. For another instance, some malaria medication produced by Chinese manufacturers have not yet passed the prequalification (PQ) of medicines stipulated by the WHO and thus was prohibited to enter Tanzania. The ignorance of PQ clearance caused delays and losses in procuring for the Project (23).

*The Tanzanian ethics committee's scientific requirements needed to meet international pilot standards. For this project, both data collection and ethics review took over a year to complete (Funder-1, BMGF, China)*.

#### 3.4.3. Internal coordination among multiple stakeholders

Participants reported that Phase I lacked timely internal communication mechanisms and failed to establish an online digital platform for decision-making and training. The decision-making mechanism of Phase I often required a work report from the local PIA to the PMO, a monthly communication meeting for reports and discussion, and the PMO's submission of an email to confirm the decision at the quarterly progress meeting. This long communication chain and the complexity of the subjects involved greatly increased the costs of communication and reduced the efficiency of implementing the project.

Under the leadership of the BMGF, Coordination Management Committee (CMC), a multilateral coordination platform, was established in Phase II to integrate all key stakeholders into one platform to promote timely decision-making and communication. In addition, WHO's external evaluation experts also coordinated communication between IHI and the Tanzanian Ministry of Health. Despite the establishment of CMC, the internal coordination of Phase II has still been chanllenged by increased scope and number of intervention sites.

#### 3.4.4. Adaptation to evolving environment

According to participants, due to the emergence of COVID-19 in recent years, the global economy has regressed, and the international community has provided less funding and supplies for malaria control in Tanzania, resulting in the loss of malaria control staff and the stagnation of the Project. In addition, border closures caused by COVID-19 impeded the cross-regional exchange of experts between China and Tanzania.

*The State Administration of Foreign Exchange had great restrictions (on foreign funding) before. The allocation was less restricted in terms of the amount after the second half of 2017, but the whole allocation process was relatively slow (PIA-financial, NIPD, China)*.

*During the COVID-19 pandemic, news reports of lockdowns in villages and deaths led to intense fear of cross-infection. Public concerns arose when they had to be regularly tested and vaccinated for malaria. Additionally, there is the complication of getting commodities, buying commodities, or dissemination. Those are about fear among the communities in a complicated way (Evaluator-3, WHO, Tanzania)*.

Due to the absence of flexible risk management scheme, which can be used to identify, assess and plan for the arrangements in work activities, financial flows and personnel deployments in advance of emergencies, the project has been impacted significantly by the pandemic and had to be suspended for several times.

### 3.5. Perceived challenges in future project design

#### 3.5.1. Inadequacy of intergovernmental dialogue

Participants expressed the expectation that the Tanzanian government could have been more directly and proactively involved in the Project. Throughout the Project's lifespan, from planning and management to the implementation and promotion process, however, high-level governmental dialogue was rarely observed, which thus delayed the institutional dissemination of project experience and outcomes. The NMCP in Tanzania was involved in Phase II, but was not directly involved in the field interventions or the provision of feedback regarding follow-up strategies.

*For interventions like this, if you are not a purely scientific research project and you have already done community interventions, you must generate dialogue between governments, such as the direct dialogue and engagement between the Chinese and British governments in Phase I (PMO-1, NHC, China)*.

#### 3.5.2. Limitations of the logistical arrangements

Participants widely expressed that the Chinese government's support for global health projects is still hampered by insufficiently designed policies and a lack of logistical incentives. Participants in the Project argued that they had to grapple with challenges tied to obtaining entry visas, the short visa durations licensed, and concerns surrounding personal security during international deployments. The issue of talent attrition within the global health landscape is steadily intensifying, and it's becoming increasingly evident that there is a paucity of career prospects for Chinese professionals dedicated to malaria control. Furthermore, opportunities in foreign DAH projects lack the attractive incentives that professionals often seek, particularly in terms of competitive salaries and avenues for career advancement and promotions. This has led to a growing concern among potential Chinese candidates about the long-term viability and desirability of pursuing careers in global health initiatives.

*Our personnel cannot come to the sites in Tanzania even if they have strong willing to do so because it takes 2–3 months to get a visa. There is not adequate security protection for our personnel to work in Tanzania (PIA-3, NIPD, China)*.

## 4. Discussion

### 4.1. Findings

This study found that identifying contextual diversities laid the foundation for formulating and tailoring global health intervention strategies. Several factors contribute to the sustainability and success of the Project, including the localization of the transferred techniques and experiences, the team-building and self-learning of implementers, the sustainability of multisource funding, and the maintenance of international partnerships. Conversely, the Project was constrained by the inadequacy of intergovernmental dialogue and limitations of the logistical arrangements.

The localized 1,7-mRCTR as a facilitator of the Project reflects the findings of other malaria-related studies. It is noted that epidemiological, ecological, and socioeconomic differences in the impacts of malaria between China and Tanzania necessitate the local adaptation of malaria control strategies for use by Chinese experts. The 1-3-7 strategy faces significant challenges when it comes to its implementation in Tanzania. This strategy was designed to revolve around swift reporting and response to cases, involving a one-day initial diagnosis through a sophisticated electronic information system, followed by a series of tests (including blood smear by microscopy, Polymerase Chain Reaction), and epidemiological investigations, all to be completed within three days. The final step involves foci investigation, treatment, and vector control, which should be carried out within seven days. Successful execution of the 1-3-7 strategy hinges on having an ample pool of skilled human resources and robust laboratory testing capabilities (12).

However, Tanzania faces a high disease incidence rate and grapples with inadequate human resources and limited laboratory testing capacity. Given these challenges, Tanzania may find it more feasible to adopt the 1,7-mRCTR approach, emphasizing prompt screening and reporting within 1 day and ensuring that patients receive treatment within seven days. This streamlined version of the strategy may align better with Tanzania's current resources and capabilities. Participants in our study revealed that 1,7-mRCTR played an active role in complementing the original NMSP in Tanzania through setting up procedural routines in high-incidence areas. The outcomes of the Project have now been supported by NMCP, and a scale-up proposal has been submitted to the Tanzanian government. This situation serves as a positive signal and recognition of the Project.

In the history of malaria elimination in China, Chinese experts have not only used a combination of measures, including MDA, indoor residual spraying (IRS), and insecticide-treated nets (ITNs), but have also adapted malaria control strategies to various local contexts based on the intensity of malaria transmission, regional stratification, vector behavior, local health infrastructure, and environmental conditions (24). These measures have been adopted and localized in Tanzania and combined with 1-7mRCTR for antimalarial purposes.

One challenge faced by the Project was the fact that the lack of involvement of the Tanzanian government led to the inadequacy of high-level intergovernmental dialogue. Experience with schistosomiasis control in China and the medical teams that China sent to Sierra Leone to support Ebola control both highlighted the role of active political will and commitment to infectious disease control as important prerequisites (25, 26). Top-down policy design and institutional guidance serve as essential components of malaria control (27). This finding also has crucial implications for the further expansion of China-Africa malaria control programmes.

**Figure 2:**
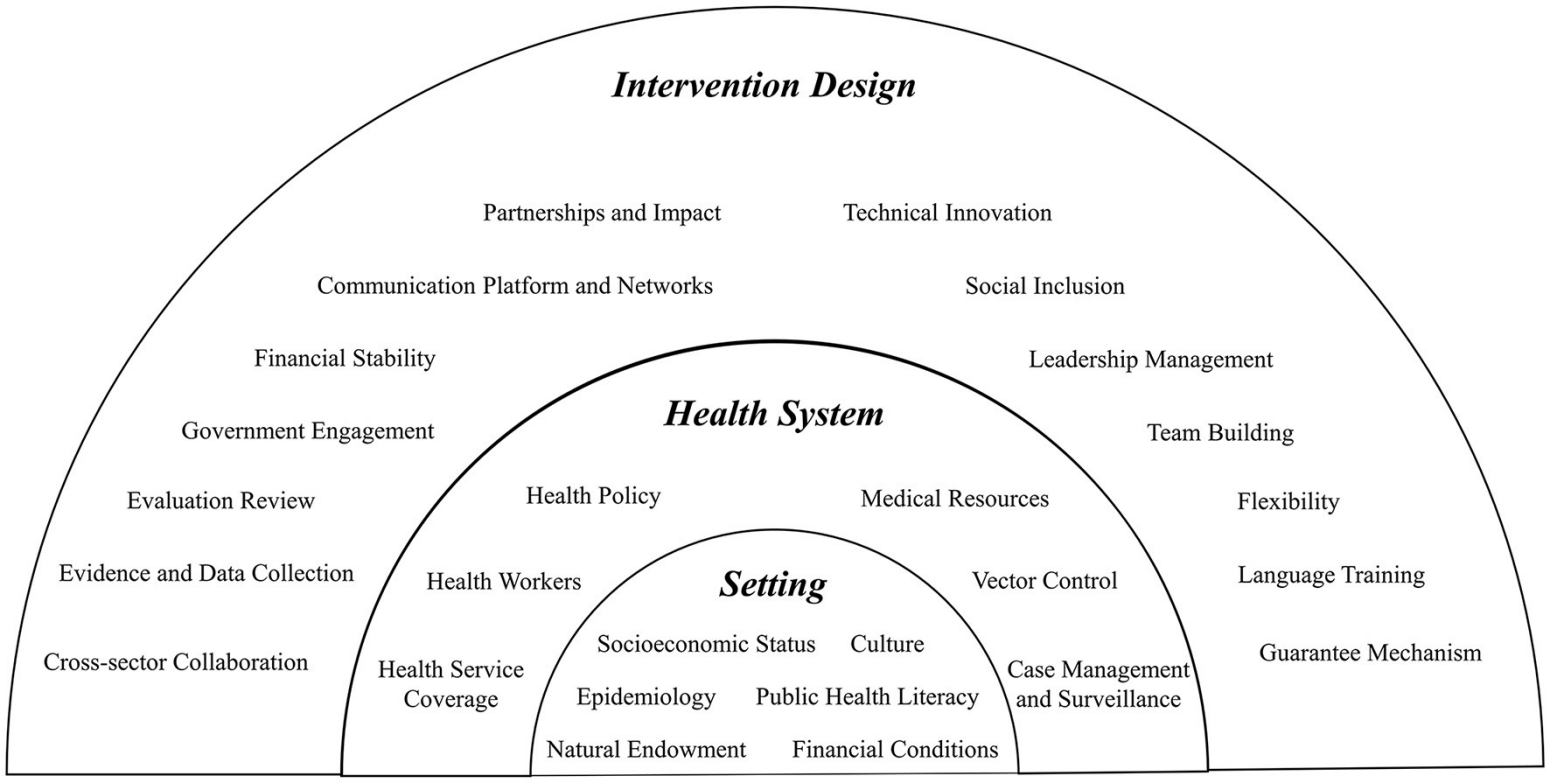
Proposed checklist for global health projects on malaria control. The project design is divided into three levels, with each of which as foundations for the higher level. Fully understanding of the intervention settings should include careful investigation of the socioeconomic status, epidemiological characteristics, natural endowment, culture, public health literacy, and financial conditions. Based on overall knowledge in setting, the project should dive deeper into the local health system with a well-rounded examination of local health policy, health workers, healthcare service coverage, and a malaria-specific study of related medical resources, vector control measures, and case management and surveillance system. Following these two steps, an intervention design can be drawn from these 14 aspects that guide health system intervention in resource-limited settings.

Based on the findings, an implementation checklist has been proposed for the design of future global health projects aimed at malaria control (Figure 2).

### 4.2. Implications and prospects for future research

Malaria continues to be a significant threat to global public health, with a disproportionately high prevalence in the African region. China, recognized for its effective malaria elimination model and as a prominent representative of the Global South, is poised to play a pivotal role in the realm of global health. The presented project serves as a sustainable endeavor aimed at advancing China's DAH, particularly within the domain of global malaria control.

The Project our article evaluated has yielded remarkable results in alleviating the burden of the disease, enhancing public health awareness, and equipping local healthcare personnel with vital skills. Furthermore, it has provided valuable insights into the complexities of crafting context-specific intervention strategies and ensuring the enduring impact of such projects within the framework of the local healthcare system.

In response to the barriers and facilitators elucidated in this study, the current emphasis for exporting antimalarial expertise must pivot toward conducting pre-implementation environmental assessments and tailored program design. Additionally, risk management structures, high-level government engagements, sustainable funding mechanisms, strategic media exposure, and the delivery of high-quality healthcare products emerge as crucial priorities.

Phase I highlighted the need to adapt interventions to the local context and to be wary of financial risks due to different financial systems. Phase I laid the groundwork for the scale-up and evidence collection in Phase II which efficiently optimized the lengthy organizational communication mechanisms and adopted a flat communication platform. Abundant lessons were learned in the Project as summarized in Table 5, particularly in Phase II where a risk management mechanism, high-level intergovernmental dialogue, sustainable financial flows, and high-quality export products were highlighted.

**Table 5:** Implications and lessons for future China global health development.

**Items**	**Recommendation**
*Comprehensive due diligence on both the epidemiological technical side and socioeconomic side*	China should maintain its current achievements in the fight against malaria in Africa. To this end, China needs to continue to maintain good international partnerships with African countries, send global health talents to Africa, strengthen the skills training of local grassroots health workers, and continue to promote the transformation of China's anti-malarial technology experience in Africa. China can take the initiative to enhance high-level intergovernmental dialogues, participate in global health diplomacy, and open the first gate of global health projects through political incentives in the form of memorandum of understanding and economic incentives in the form of reduced tariffs to help the global South countries deepen cooperation. China's anti-malaria experience can be exported as a global public good in the form of diagnostic methods, treatment methods, and anti-malarial drugs to alleviate the status quo of global health inequalities. Countries with high malaria incidence need to be vigilant against internal corruption and misappropriation of public health funds and fully invest international DAH institutions and funds in malaria control, a low-cost, high-yield health initiative.
*Context-based practices and project design*	
*Sustainability of national funding on global health and global development aid*	
*Dynamic management mechanism and competent team building*	China should improve its global health project management system and establish proper logistics arrangements for global health workforce. More efforts are in need to train all-around global health talents in the fields of epidemiology, project management, and foreign affairs. China should explore its paradigm of cooperation in global health projects, provide novel perspectives and a new foothold for the Global South countries, and realize a dedicated and dynamic management pathway in global health projects.
*Engagement in high-level inter-governmental discussion*	China should actively engage in dialogue with active actors in global health by drawing timely academic output and media coverage. By holding lectures, publishing articles, news reports, etc., China's theories, and technologies will be shown to the international community. China could listen to the inconsistent voices of the world, so as to improve the existing problems and gain stronger evidence-based practice in international health collaboration. China can also understand the effective institutional characteristics and organizational models of other countries, to facilitate the localization of China's practical experience and achieve capacity building and improve the self-sustained health system for recipient countries.
*Exposure to media and academia concerning international visibility and attractiveness*	
*International cooperation of public health products, technologies and services in line with international standards*	Along with the institutionalized establishment of the China-Africa Malaria Control Program subsequently, China can gradually level its responsibility and role to eventually become a key donor country in global health. In recent years, the Chinese government has increased its involvement in global health. In the process of exporting anti-malaria experienced professional teams and medical supplies to foreign countries, China may gain more experience in foreign public health engagement from its cooperation with different countries, which contributes to the advancement of global health.

Inspired by the positive outcomes from the Project and the strengthening bonds between China and Africa cooperation in the fight against malaria, the United Nations Peace Fund, the World Health Organization's Global Malaria Initiative, in conjunction with Special Programme for Research and Training in Tropical Diseases extended their support through further funding for the NIPD to carry out the Strengthening the Capacity of Surveillance and Response (SCSR) project. The SCSR aims to promote the expansion of 1,7-mRCTR and other related interventions to Tanzania, Zambia, Burkina Faso, and Senegal (28). Until 2023, the SCSR project has significantly bolstered Tanzania's ability to monitor and respond to malaria cases while maintaining the achievements of the original project. The sustained funding from the SCSR has also played a crucial role in ensuring the project's long-term viability and impact.

### 4.3. Strengths and limitations

This study encounters several limitations. Firstly, due to constraints such as limited research funding as well as human and material resources, not all stakeholders in the Project were included in the interviews; most of the participants were drawn from the Chinese intervention team, with fewer interviews conducted from the perspective of Tanzanians as recipients. Secondly, the Hawthorne effect occasionally proved challenging to avoid during the interviews. Consequently, there is possibility that the researcher may have inadvertently exaggerated or narrowed certain truths, potentially distorting the importance of specific influencing factors (29). Thirdly, the interviews relied on self-reporting by the interviewees and thus limited by social desirability bias (30). Participants might not have been able to fully identify the challenges faced by the current project due to their current positions and social pressures.

This study offers valuable insights to the existing literature. Firstly, although China has executed numerous aid projects involving the deployment of medical teams abroad, it has relatively limited experience in the realm of global public health and health system aid initiatives. The Project, as one of China's pioneering overseas projects, has opened up a global health network for China and provided a model for future endeavors. Additionally, the Project diverges from the traditional pattern of North–South cooperation in previous public health programs, representing a large-scale malaria control initiative that emphasizes South–South cooperation. It marks a significant milestone following China's successful elimination of malaria, symbolizing a breakthrough and China's influential role in global health.

Furthermore, considering China's indication of active participation in future global health projects, this study employs a qualitative approach to systematically review the experiences of two phases of the Project in Tanzania. It identifies disparities between Phase I and Phase II in terms of organizational characteristics and management models, summarizes current challenges in China's global health engagement, and furnishes evidence-based policy implications for the future expansion of the global health landscape.

Moreover, this study represents a further integration and improvement of two established theoretical frameworks (CFIR and RE-AIM). These two frameworks have been consolidated into a single theoretical framework, both from a process and outcome perspective, with the aim of bolstering the robustness and comprehensiveness of analysis. In addition, this study included an appropriate number of interviewees based on Grounded Theory and a scientific and rigorous sampling method; the data were coded by three researchers using NVivo to reduce the inaccuracy of single coding; pre-interviews were conducted before the formal interviews to further adapt the interview outline.

Finally, this study focused on multiple stakeholders within the Chinese team. It also considered the Tanzanian perspective on the demand side of the service, thereby making an important contribution to the existing literature on the role of China's participation in global health and providing an in-depth and systematic overview of the barriers, facilitators, future opportunities and challenges associated China-initiated global malaria control programme.

## 5. Conclusions

The Project was China's first brave step forwards in the field of global health. The COVID-19 pandemic may have put the China-Africa malaria control programme on hold for a short time, but the concept of the Health Silk Road has generated strong interest in the task of promoting DAH from active actors in global health. In contrast to previous DAH paths taken by developed countries, China has chosen to promote malaria control from a comprehensive and systematic perspective that takes into account the whole health system. To guide China's future participation and even leadership in DAH projects more effectively, the findings of this study should be considered alongside the Project's actual outcomes in local malaria control as they have been published and reported (8). The Project led to the development of context-tailored strategies to achieve outstanding outcomes through multiple approach of improving public health literacy, expanding the primary health care team, and making the health care system more resilient. The Project also provided a grand platform that Chinese and Tanzanian personnel can use to learn from and exchange expertise with each other, thereby deepening the partnership between the two countries.

Hitherto, the China-Africa Malaria Control Project has been scaled up to four countries in Africa. In the hope of providing the cutting-edge experience and technical expertise to all high malaria-endemic countries in Africa to use as a reference, the need for China to carefully face the two challenges of funding gaps and lack of support from recipient governments remains ongoing. Due to the development of international structures, the stability of global health projects has frequently been questioned. It is recommended that China should form an institutionalized DAH system as soon as possible, establish a sustainable pool of development assistance funds, and guarantee the steady progress of global health projects.

## Data availability statement

The original contributions presented in the study are included in the article/Supplementary material, further inquiries can be directed to the corresponding authors.

## Ethics statement

The studies involving human participants were reviewed and approved by the Ethical Review Committee of the Chinese Center for Disease Control and Prevention (No. 20190115). Written informed consent from the participants was not required to participate in this study in accordance with the national legislation and the institutional requirement.

## Author contributions

ZS, HZho, FC, HL, EW, and ZT designed and planned the study with input from XZha, DW, FY, and SL. ZS, HZho, FC, HL, EW, HZha, and XZha collected the data. ZS, HZho, and FC led the analysis with the input of all authors. ZS, HZho, and FC wrote the manuscript with input from XZha, DW, FY, and XZho. All authors have read and approved the final manuscript.
