# Supplementary material for: Understanding the China-Tanzania Malaria Control Project: lessons learned from a multi-stakeholder qualitative study
Source: Front Public Health. 2023 Sep 21;11:1229675. doi: 10.3389/fpubh.2023.1229675 (PMC10552642; doi:10.3389/fpubh.2023.1229675)
Supplement: Supplementary file 1 [file Data_Sheet_1.DOCX]

**Understanding the China-Tanzania Malaria Control Project:**

**Lessons Learned from a Multi-Stakeholder Qualitative Study**

***Interviewee: Evaluation Experts***

1. Please briefly describe your background, including education, title, specialty, and professional experience, and your role in the project (involvement in phase1/2/both).
2. What data resources have you relied on for your evaluation of this project?
3. To what extent do you think the implementation measures are appropriate to the malaria situation in the pilot areas (e.g. Rufiji, Kilwa)?
4. How did you divide the work between Tanzanian health workers and the Chinese implementation team on the site?

- Prompt: Is there any challenge or advantage when cooperating with the Chinese implementation team?
- Prompt: (If involve in both phases)Do you see any changes in the project management process between phase 1 and phase 2? (Including but not limited to fund management and use, project implementation measures, staff scheduling and arrangements) Do you see these changes as improvement or new challenges?

1. How would you evaluate the introduction of the 1-7mRCTR strategy in Tanzania? Does it fit with the national malaria strategic plan? What benefits did it bring about? What was the main concern about its introduction?

- Prompt: How did the 1-7mRCTR integrate into the local health system?
- Prompt: Is there any barrier when putting forward 1-7mRCTR? (e.g. Financial and time costs? Human resources allocation? Motivation of local citizens?)

1. What do you think are the main differences between the 3T strategy brought about by WHO and the 1-3-7 strategy of China? Given the implementation outcomes, which of them best fit in the Tanzanian context and why?
2. Did you evaluate feedback from local citizens and local governments? How did local citizens and local governments support the overall China-Tanzania project on malaria control?

- Prompt: What factors do you think have influenced the perception of the project by local people and local authorities?

1. Besides what was mentioned above, what are the main enablers and challenges of the China-Tanzania project outcomes?
2. To what extent was the Tanzanian government involved in supporting or promoting the implementation?

- Prompt: Was the Tanzanian government engaged in the pilot phase or the demonstration phase? Which governmental agencies were involved?

1. Compared to previous partners in malaria control in Tanzania (for example global fund, U.S. president initiative), what was this project's added value to the national malaria control plan?

- Prompt: What did it bring about to local people and organizations?

1. How do you evaluate the sustainability of the project? How do you evaluate the overall cost-effectiveness of this project?

- Prompt: Would local institutions in Tanzania further adopt and continue the measures (for example 1-7 strategy, barefoot doctors) on malaria control?

1. What are the present barriers to malaria control breakthroughs in Tanzania? From your experience and professional understanding, what will be the most needed factors in Tanzania's malaria control programme?

**Understanding the China-Tanzania Malaria Control Project:**

**Lessons Learned from a Multi-Stakeholder Qualitative Study**

***Supply side- Finance Funder***

***Interviewee: Funders [i.e. DFID, BMGF]***

1. Please give a brief description of your background, including education, title, specialty, and years of experience.
2. How did you engage in the Project? What role did you play? What was your main responsibility? What was the organizational structure inside the funding organization? [Individuals Involved]
3. How was the relationship among funding organization and other implementation organizations? [Inner Setting]

- Prompt: How was the communication mode or form among funding and implementation organizations?

1. How was the general finance flow of the Project? [Implementation Characteristics]

- Prompt: What is the process of setting out the budget, distributing the funding, submitting the settlement reports during the Project?
- Prompt: What problem or obstacle was introduced in the funding process? How do the funding organization and implementation organization solve the problem?
- Prompt: What are the experiences and lessons learned that can be shared with other global health cooperation and funding projects? What are the suggestions for improvement for the future projects?

1. In your view, to what extent can the Project be scaled up to the whole Tanzania or be contributed to national health plans? [Reach / Effectiveness]

- Prompt for: contextual factors, e.g. COVID-19; natural factors (natural environment, vector species, etc.); social factors (social acceptance, behaviors, etc); policy factors (national and local policies relating to malaria, but also health system organization, drug /medical commodity availability, etc.); institutional factors (levels of capacity and resources at local level / in different places, presence of systems for e.g. data reporting, etc.); availability of resources; political support, recognition of personnel, fidelity of implementation, etc.health care related departments?

1. In your understanding, how do you think of the overall funding process of the Project? How does the funding different from those used by the main global malaria programs (e.g., Roll Back Malaria, the Global Fund , and the President’s Malaria Initiative)? [Intervention Characteristics]

- Prompt: Have your initial motives of funding reach its end in the Project? How do you think of the funding process and its outcome, from the aspect of safety and effectiveness? Is the process of funding transparent enough for the organization? Have you encountered any irregular operation in the funding process? [Reach / Effectiveness]
- Prompt: In the Tanzanian project, is there any difference between the mode of managing and using funds and the common international mode? Is the flexibility of funds higher or lower than other projects? [Adoption / Implementation]

1. Given the Chinese government’s intention to support greater health cooperation with developing countries, what are the priorities or the main concern points for funding organizations to invest in the field of global health? [Inner Setting]

- Capacity, prompt for: the capacity of researchers/universities to play a more prominent role in global health; what opportunities are needed to enable young cohort of researchers, etc. to gain experience of working in low-income countries; funding from Chinese government to provide more significant support to global health; language; project management skills of technical agencies (e.g. CDC) / universities.
- Systems, prompt for: greater support from Chinese government (NHC, CIDCA, other?); more apparent funding channels for Chinese agencies to work with overseas counterparts on this kind of intervention…

**Understanding the China-Tanzania Malaria Control Project:**

**Lessons Learned from a Multi-Stakeholder Qualitative Study**

**Interview Guides**

**Supply side- Implementation**

***Interviewee: Project Implementation Agencies (PIAs - China NIPD)***

1. Please give a brief description of your background, including education, title, specialty, and years of experience.
2. How did you engage in the Project? What role did you play? What was your main responsibility? What was the organizational structure inside PIAS? [Individuals Involved]
3. What is your daily task for the Project? How do you evaluate your task completion in the Project? Do you feel that your work is achieving the desired results? [Individual involved]

- Prompt: What difficulties have you encountered and how did you solve them? Did you get help from other people? (e.g.from Tanzanian staff, technical training)

1. How was the relationship among PIAs (/your organization) and other project-related organizations? [Inner Setting]

- Prompt: How was the communication mode or form among project cooperative organizations? How do you cooperate with Tanzanian staff ? (division of work, Feedback form, communication effect)
- Prompt: What was the general workflow of the Project? (e.g. equipment, finance, personnel)

1. Could you sort out the main work PIAs (/your organization) have done to support or promote the implementation? [Implementation Characteristics / Process]

- Prompt: How do you view the significance of PIAs (/your organization) to the Project?
- Prompt: How was the process of the Project? Could you list a timeline?
- Prompt: In the process of the project, how do the staff of the relevant departments view the project, and what is the attitude and motivation of the staff? (agree, need, cooperate with hospital action)
- (Specifically problem about staff and resource) Prompt for: Is there a guarantee mechanism for the exit or entry of relevant personnel? How to raise relevant materials? According to what criteria are relevant materials allocated? In the financial system, who is in charge of the distribution of money and goods? How to ensure the safety of funds? How to evaluate the benefits of material use?

1. In specific, how was the 1,7-mRCTR designed? [Implementation Characteristics]

- Prompt: Could you please give a brief introduction about the background of its introduction to the Project?
- Prompt: Could you please share the experience and process in transforming the 1-3-7 approach in Chinese experience to the 1,7-mRCTR in the Project, making it more suitable for Tanzania?

1. How did the Project integrate 1,7-mRCTR into the local medical practice? What adjustments have been made on the initial model to adapt to the local practice? [Process / Implementation]
2. What factors facilitated/hindered the implementation of the Project? [Implementation Characteristics]

- Prompt: Did you have sufficient resources and policy support (including support from the health care commission, hospital, community, etc.) in the process of promoting the Project? [Outer Setting]
- Prompt: If not, which kind of support do you need in the implementation? What would you do to solve the problem?

1. What outcomes did the Project bring about? What did it bring about to local people and organizations? Is there any positive or negative effect? What kind of change can it bring to you personally and to your unit? (income, career gain, healer-patient relationship, reputation of the area) [Process / Effectiveness]

- Prompt: In your understanding, to what extent does the pilot display a distinctive approach to malaria control and elimination, different to those used by the major global malaria programs (e.g. Roll Back Malaria, the Global Fund and the President’s Malaria Initiative)?
- Prompt: Please explain the purpose and achievements of the second phase of the Project, supported by the Gates Foundation. To what extent has this phase met its aims? What makes it different from the first phase?

1. In your view, to what extent can the Project be scaled up to the whole Tanzania or be contributed to national health plans? [Reach / Effectiveness]

- Prompt for: contextual factors, e.g. COVID-19; natural factors (natural environment, vector species, etc.); social factors (social acceptance, behaviors, etc); policy factors (national and local policies relating to malaria, but also health system organization, drug /medical commodity availability, etc.); institutional factors (levels of capacity and resources at local level / in different places, presence of systems for e.g. data reporting, etc.); availability of resources; political support, recognition of personnel, fidelity of implementation, etc.health care related departments?

1. When some Chinese experts withdrew from the project, what challenges has the Project faced? What did that bring about to the implementation and personnel? How did you / your organization solve the difficulties? [Maintenance]
2. To what extent did the Project potentially provide a model for future cooperation between China and African countries in malaria control/elimination? How to promote the model?[Adoption / Implementation]

- Prompt: Please explain your response. Why does this (or does this not) provide a model for future collaboration? Is there support for this within Tanzanian government?
- Prompt: What are the experiences and lessons learned that can be shared with other projects on malaria control? Do you have any suggestion for improvement for the future development of projects on malaria control? How could those limitations be addressed?

**Understanding the China-Tanzania Malaria Control Project:**

**Lessons Learned from a Multi-Stakeholder Qualitative Study**

**Supply side- Policy & Management**

***Interviewee: Project Management Office (PMO)***

1. Please give a brief description of your background, including education, title, specialty, and years of experience.
2. How did you engage in the Project? What role did you play? What were your main responsibility? What was the organizational structure inside PMO? [Individuals Involved]
3. How was the relationship among PMO and other project-related organizations?

[Inner Setting]

- Prompt: How was the communication mode or form among project cooperative organizations?
- Prompt: What was the general workflow of the Project? (e.g. equipment, finance, personnel)

1. What were the main goals or targets of the Project? [Outer Setting / Inner Setting]

- Prompt: From your understanding, to what extent did the Project fulfill the initial goals?
- Prompt: What caused the alignment or the gap between the goals and outcomes? (specific reasons or factors or events)
- Prompt: (If gap) What have PMO done to eliminate the gap and promote a better implementation of the Project?

1. Could you sort out the main work PMO has done to support or promote the implementation? [Implementation Characteristics / Process]

- Prompt: How do you view the significance of PMO to the Project?
- Prompt: How was the process of the Project? Could you list a timeline?

1. From your understanding, what factors facilitated/hindered the implementation of the Project? [Implementation Characteristics]

- Prompt: (If hindered) Was there any barriers or regulations of local systems in pilot areas or countries that hindered the implementation of the project? Have there been enough regulations and plans that could guide relevant staff in executing implementation (e.g. data reporting, quality review and feedback, education and training activities for medical and nursing staff)?[Outer Setting]
- Prompt: (If hindered) What did you/PMO do to solve the barriers?

1. What outcomes did the Project bring about? What did it bring about to local people and organizations? Is there any positive or negative effect? In your view, to what extent can the Project be scaled up to the whole Tanzania or be contributed to national health plans? [Reach / Effectiveness]
2. How do you evaluate the sustainability of the Project? [Adoption / Maintenance]

- Prompt: Did you or your organization take attempt to promote its continuity and adaptability afterwards?
- Prompt: Did local institutions further adopt the measures on malaria control after the Project?

1. To what extent did the Project potentially provide a model for future cooperation between China and African countries in malaria control/elimination? How to promote the model?[Adoption / Implementation]

- Prompt: What are the limitations of this Project in providing a model for future cooperation? How could those limitations be addressed?
- Prompt: Given the Chinese government’s intention to support greater health cooperation with developing countries, what are the priorities or the main concerns for relevant health agencies to play a larger role in the field of global health?
- Prompt: How do you view the effect of the Project on the relationship between China and Africa?
