# Supplementary material for: Understanding the China-Tanzania Malaria Control Project: lessons learned from a multi-stakeholder qualitative study
Source: Front Public Health. 2023 Sep 21;11:1229675. doi: 10.3389/fpubh.2023.1229675 (PMC10552642; doi:10.3389/fpubh.2023.1229675)
Supplement: Supplementary file 2 [file Data_Sheet_2.DOCX]

**Codebook**

**1. Codebook for CFIR**

| I. Intervention characteristics |  |
| --- | --- |
| A. Intervention sources | Definition: It refers to the various types of resources (more specific) related to the intervention owned or lacking by the intervention implementer. |
|  |  |
|  | Inclusion Criteria: Include statements about the resources (more specific) owned or lacking of the intervention of intervention implementer, e.g., some resources (monitoring system) to facilitate the implementation of the intervention. |
|  |  |
|  | [Exclusion Criteria: Exclude some resources that focus on experience because they have been encoded into Available Resources.](http://cfirwiki.net/wiki/index.php?title=Available_Resources) |
|  |  |
| B. Complexity | Definition: Perceived difficulty of the intervention, reflected by duration, scope, radicalness, disruptiveness, centrality, and intricacy and number of steps required to implement. |
|  |  |
|  | Inclusion Criteria: Code statements regarding the complexity of the intervention itself. Refers to some different or defective factors of the intervened party, which are related to specific interventions and often make the implementation of the intervention more difficult. |
|  |  |
|  | [Exclusion Criteria: Exclude statements regarding the complexity of implementation and code to the appropriate CFIR code, e.g., difficulties related to space are coded to Available Resources and difficulties related to engaging participants in a new program are coded to Engaging.](http://cfirwiki.net/wiki/index.php?title=Engaging) |
|  |  |
| C. Financial stability | Definition: Funds of the intervention and funds associated with implementing the intervention including investment, supply, and opportunity costs. |
|  |  |
|  | Inclusion Criteria: Include statements related to the funds and the stability of the intervention and its implementation. |
|  |  |
|  | [Exclusion Criteria: Exclude statements related to physical space and time, and code to Available resources.](http://cfirwiki.net/wiki/index.php?title=Available_Resources) |
| **II. Process** |  |
| D. Planning | Definition: The degree to which a scheme or method of behavior and tasks for implementing an intervention are developed in advance, and the quality of those schemes or methods. |
|  |  |
|  | Inclusion Criteria: Include evidence of pre-implementation diagnostic assessments and planning, as well as refinements to the plan. |
|  |  |
| E. Engaging | Definition: Attracting and involving appropriate individuals in the implementation and use of the intervention through a combined strategy of social marketing, education, role modeling, training, and other similar activities. |
|  |  |
|  | Inclusion Criteria: Include statements related to engagement strategies and outcomes, i.e., if and how intervention participants became engaged with the intervention and what their role is in implementation. In addition, you may also want to code the "quality" of staff - their capabilities, motivation, and skills, i.e., how good they are at their job, and this data affects the rating as well. Note some obvious sub-constructs of Engaging, including Opinion Leaders, Internal Appointed Leaders, External Change Agents, Key Stakeholders. |
|  |  |
|  | **Note: Although both strategies and outcomes are coded here, the outcome of engagement efforts determines the rating, i.e., if there are repeated attempts to engage staff that are unsuccessful, or if a role is vacant, the construct receives a negative rating.* |
|  |  |
|  | Exclusion Criteria: Exclude statements related to exact actions or interventions implemented, and code to Executing. Exclude or double the statements related to evaluation of intervention participants, i.e., participants failed to realize their responsibilities and duties during the intervention to Reflecting & evaluating. |
|  |  |
| F. Executing | Definition: Carrying out or accomplishing the implementation according to plan. |
|  | Inclusion Criteria: Include statements that demonstrate how implementation occurred with respect to the implementation plan. |
|  | *Note: Executing is coded very infrequently due to a lack of planning. However, some studies have used fidelity measures to assess executing, as an indication of the degree to which implementation was accomplished according to plan. |
|  | Exclusion Criteria: |
| G. Reflecting & evaluating | Definition: Quantitative and qualitative feedback about the progress and quality of implementation accompanied by regular personal and team debriefing about progress and experience. |
|  |  |
|  | Inclusion Criteria: Include statements that refer to the implementation team’s (lack of) assessment of the progress toward and impact of implementation, as well as the interpretation of outcomes related to implementation. Reflecting and Evaluating is part of the implementation process; it likely ends when implementation activities end. It does not require goals to be explicitly articulated; it can focus on descriptions of the current state with real-time judgment, though there may be an implied goal (e.g., we need to implement the intervention) when the implementation team discusses feedback in terms of adjustments needed to complete implementation. |
|  | [Exclusion Criteria: Exclude statements related to the (lack of) alignment of implementation and intervention goals with larger organizational goals, as well as feedback to staff regarding those goals, e.g., regular audit and feedback showing any gaps between the current organizational status and the goal, and code to Goals & Feedback. Goals and Feedback include organizational processes and supporting structures independent of the implementation process. Evidence of the integration of evaluation components used as part of “Reflecting and Evaluating” into on-going or sustained organizational structures and processes may be (double) coded to Goals and feedback.](http://cfirwiki.net/wiki/index.php?title=Goals_&_Feedback) |
|  | [Exclude statements that capture reflecting and evaluating that participants may do during the interview, for example, related to the success of the implementation, and code to Knowledge & beliefs about the intervention.](http://cfirwiki.net/wiki/index.php?title=Knowledge_&_Beliefs_about_the_Intervention) |
| Ⅲ. Individuals Involved |  |
| H. Knowledge & beliefs | Definition: Individuals’ attitudes toward and value placed on the intervention, as well as familiarity with facts, truths, and principles related to the intervention. |
|  |  |
|  | Inclusion Criteria: Personal professional knowledge background, work experience, confidence in the success of the project. |
|  |  |
|  |  |
| I. Self-efficacy | Definition: Individual belief in their own capabilities to execute courses of action to achieve implementation goals. |
|  |  |
|  | Inclusion Criteria: In the process of intervention implementation, the individual's confidence of completion and grasp of the intervention. |
|  |  |
|  | [Exclusion Criteria: Individual statements about self-change during intervention implementation should be coded into Individual stages of Change.](https://cfirguide.org/) |
|  |  |
| J. Individual stage of change | Definition: Characterization of the phase an individual is in, as s/he progresses toward skilled, enthusiastic, and sustained use of the innovation. |
|  |  |
|  | Inclusion Criteria: It focuses on the change in individual attitude, knowledge and ability, which is generally positive. e.g. Individuals become more proficient or gain other skills. |
|  |  |
|  | [Exclusion Criteria: Individual statements about confidence and beliefs of the project should be coded into Knowledge & beliefs.](https://cfirguide.org/) |
|  |  |
| K. Identification with organization | Definition: A broad construct related to how individuals perceive the organization, and their relationship and degree of commitment with that organization. |
|  |  |
|  | Inclusion Criteria: Recognition of the organization's contribution and the impact it has had. |
|  |  |
|  | [Exclusion Criteria: Exclude statements related to the evaluation of the organization during project implementation. e.g. Suggestions for improvement of organizational deficiencies should be coded into Reflecting and evaluating.](https://cfirguide.org/) |
| **Ⅳ. Outer Setting** |  |
| L. Needs & resources of those served by the organization | Definition: The extent to which the needs of those served by the organization (e.g., patients, local health facilities), as well as barriers and facilitators to meet those needs, are accurately known and prioritized by the organization. |
|  |  |
|  | Inclusion Criteria: Include statements demonstrating (lack of) awareness of the needs and resources of those served by the organization. Analysts may be able to infer the level of awareness based on statements about: 1. Perceived need for the project implementation based on the needs of those served by the organization; 2. Barriers and facilitators of those served by the organization to participating in the implementation; 3. Participant feedback on the intervention, i.e., satisfaction and success in a program. In addition, include statements that capture whether or not awareness of the needs and resources of those served by the organization influenced the implementation or adaptation of the innovation. |
|  |  |
|  | Exclusion Criteria: Exclude statements that demonstrate a strong need for the innovation and/or that the current situation is untenable and code to Tension for Change. Exclude statements related to engagement strategies and outcomes, e.g., how organization participants became engaged with the intervention, and code to Engaging. |
|  |  |
| M. Cosmopolitanism | Definition: Reflections and suggestions on global health cooperation projects. The degree to which an organization is networked with other external organizations. |
|  |  |
|  | Inclusion Criteria: Include some suggestions on international cooperation and globalization and descriptions of outside group memberships and initiatives for global health cooperation. |
|  |  |
|  | Exclusion Criteria: Exclude statements about general networking, communication, and relationships in the organization, such as descriptions of meetings, email groups, or other methods of keeping people connected and informed, and statements related to team formation, quality, and functioning, and code to Networks & Communications. |
|  |  |
| N. Organizational networks | Definition: The construction and staffing of local medical and health institutions in the pilot areas, as well as the contacts between the various medical and health institutions and the government. |
|  |  |
|  | Inclusion Criteria: The statement of the functions of local health institutions and their interactions on the implementation of the project. |
|  |  |
|  | Exclusion Criteria: Excluded statements related to interactions between Chinese institutions, other international institutions (non-local institutions) are excluded and should be coded into External networks & communications. |
|  |  |
| O. External networks & communications | Definition: The nature and quality of organization networks and communications in non-pilot areas. |
|  |  |
|  | Inclusion Criteria: Include statements about general networking, communication, and relationships in the organization, such as descriptions of meetings, email groups, or other methods of keeping people connected and informed, and statements related to team formation, quality, and functioning. |
|  |  |
|  | Exclusion Criteria: Exclude statements related to personal professional knowledge background, work experience, confidence in the success of the project and code to Knowledge & beliefs. Exclude statements related to engagement strategies and outcomes, e.g., how key stakeholders became engaged with the innovation and what their role is in implementation, and code to Engaging. |
|  |  |
| P. External policy & incentives | Definition: A broad construct that includes external strategies to spread innovations including policy and regulations (governmental or other central entity), external mandates, recommendations and guidelines, pay-for-performance, collaboration, and public or benchmark reporting. |
|  |  |
|  | Inclusion Criteria: Include descriptions of external performance measures from the system and statements related to international policy support and funding. |
|  |  |
| **Ⅴ. Inner setting** |  |
| Q. Structural characteristics | Definition: The social architecture, age, maturity, and size of an organization. |
|  |  |
| R. Networks & communications | Definition: The nature and quality of webs of social networks, and the nature and quality of formal and informal communications within an organization. |
|  |  |
|  | Inclusion Criteria: Include statements about general networking, communication, and relationships in the organization, such as descriptions of meetings, email groups, or other methods of keeping people connected and informed, and statements related to team formation, quality, and functioning. |
|  |  |
|  | Exclusion Criteria: Exclude statements related to interactions between Chinese institutions, other international institutions (non-local institutions) are excluded and should be coded into External networks & communications. |
|  |  |
| S. Available resources | Definition: The level of resources organizational dedicated for implementation and on-going operations including physical space and time. |
|  |  |
|  | Inclusion Criteria: Include some human and material resources (e.g. local medical institutions, medical personnel, sponsored supplies, etc.) |
|  |  |
|  | Exclusion Criteria: In a research study, exclude statements related to resources needed for conducting the research components (e.g., time to complete research tasks, such as IRB applications, consenting patients). |
|  |  |
|  | Exclude statements about the resources (more specific) owned or lacking of the intervention of intervention implementer, e.g., some resources (monitoring system) to facilitate the implementation of the intervention. |
|  |  |
| T. Organizational Incentives & rewards | Definition: Extrinsic incentives such as goal-sharing, awards, performance reviews, promotions, and raises in salary, and less tangible incentives such as increased stature or respect. |
|  |  |
|  | Inclusion Criteria: Include statements related to whether organizational incentive systems are in place to foster (or hinder) implementation, e.g., rewards or disincentives for staff engaging in the implementation. |
|  |  |
| U. Goals & feedback | Definition: The degree to which goals are clearly communicated, acted upon, and fed back to staff, and alignment of that feedback with goals. |
|  |  |
|  | Inclusion Criteria: Include statements related to the (lack of) alignment of implementation and intervention goals with larger organizational goals, as well as feedback to staff regarding those goals, e.g., regular audit and feedback showing any gaps between the current organizational status and the goal. Goals and Feedback include organizational processes and supporting structures independent of the implementation process. Evidence of the integration of evaluation components used as part of “Reflecting and Evaluating” into on-going or sustained organizational structures and processes may be (double) coded to Goals and feedback. |
|  |  |
| V. Leadership engagement | Definition: Commitment, involvement, and accountability of leaders and managers with the implementation of the intervention. |
|  |  |
|  | Inclusion Criteria: Include statements regarding the level of engagement of organizational leadership. |
|  |  |
|  | Exclusion Criteria: Exclude or double code statements regarding leadership engagement to Engaging if an organizational leader is also an implementation leader, e.g., if a director of primary care takes the lead in implementing a new treatment guideline. |
|  | *Note that a key characteristic of this Implementation Leader/Champion is that s/he is also an Organizational Leader.* |
|  |  |
| W. Compatibility | Definition: The degree of tangible fit between meaning and values attached to the innovation by involved individuals, how those align with individuals’ own norms, values, and perceived risks and needs, and how the innovation fits with existing workflows and systems. Organizations need adjust or formulate new policies and systems to achieve cooperation goals when they cooperate. |
|  |  |
|  | Inclusion Criteria: Include statements that demonstrate the level of compatibility the implementation has with organizational values and work processes. Include statements that the implementation did or did not need to be adapted as evidence of compatibility or lack of compatibility. |
|  |  |
|  | Exclusion Criteria: Exclude or double code statements regarding the priority of the innovation based on compatibility with organizational values to Relative Priority, e.g., if an innovation is not prioritized because it is not compatible with organizational values. |
|  |  |
| X. Learning climate | Definition: A climate in which: 1. Leaders express their own fallibility and need for team members’ assistance and input; 2. Team members feel that they are essential, valued, and knowledgeable partners in the change process; 3. Individuals feel psychologically safe to try new methods; and 4. There is sufficient time and space for reflective thinking and evaluation. 5. Ability training in the organization (such as language courses), members thus feel the improvement of their own ability. |
|  |  |
|  | Inclusion Criteria: Include statements that support (or refute) the degree to which key components of an organization exhibit a “learning climate.” |
|  |  |
| Y. Tension for change | Definition: The degree to which stakeholders perceive the current situation as intolerable or needing change. |
|  |  |
|  | Inclusion Criteria: Include statements that (do not) demonstrate a strong need for the intervention and/or that the current situation is untenable, e.g., statements that the intervention is absolutely necessary or that the intervention is redundant with other programs. |
|  |  |
|  |  |

**2. Codebook for RE-AIM**

| **1. Reach** | The absolute number, proportion, and representativeness (whether participants have characteristics that reflect the target population’s characteristics) of individuals exposed to the intervention; as well as characteristics of those who were eligible but not reached |
| --- | --- |
| A. Target population | The target population for intervention. The target population is the direct object of intervention implementation, who can often gain benefits during the intervention. |
| B. Proportion | At the site of the project implementation, a certain proportion of the target population received intervention. |
| C. Penetration | The target population can get used to intervention and have good compliance with it. |
|  |  |
| **2. Effectiveness** | The impact of an intervention on important outcomes. This includes potential negative effects, quality of life, and economic outcomes |
| D. Community health outcomes | Changes in relevant health indicators of the target population (e.g. morbidity, case fatality, etc.) during the intervention. |
| E. Quality of life | People's life expectancy, social economy, social security changes, etc |
| F. Potential negative effects | Negative effects due to project implementation. |
|  |  |
| **3. Adoption** | The absolute number, proportion, and representativeness of settings and staff who are willing to initiate a program or approve a policy |
| G. Target intervention agents | Whether the intervention was endorsed by the members of the organization, the attitude of the staff towards the project and the adoption of the intervention. |
| H. Organization penetration | To what extent the project personnel can consciously comply with the requirements and reasonably implement the intervention in the implementation process. |
|  |  |
| **4. Implementation** | To what extent is the intervention delivered as designed; includes how closely and consistently staff members follow established protocols, as well as the time and cost of the program |
| I. Adaptability | Interventions are tailored to the environment of the project site and the characteristics of the target population. |
| J. Cost | The cost (capital, manpower and material resources) invested in the implementation of the intervention and the relevant cost-benefit analysis |
| K. Consistency of delivery | To what extent the intervention was inconsistent in its implementation compared to the intended design |
|  |  |
| **4. Maintenance** | At the setting level, the extent to which a program or policy becomes part of the routine organizational practices and policies |
| L. Sustainability | Interventions can be implemented over a long period of time, and factors that affect the duration of the intervention. |
| M. Institutionalization | The institution of different countries/organizations/institutions should be adjusted, or a new institution should be developed to standardize and rationalize the intervention. |
| N. Promotion | To what extend the intervention can be extended to other regions and the factors influencing its spread. |
